# Bridging the Gap: From Biomechanics and Functional Morphology of Plants to Biomimetic Developments

**DOI:** 10.3390/biomimetics6040060

**Published:** 2021-10-18

**Authors:** Olga Speck, Thomas Speck

**Affiliations:** 1 Plant Biomechanics Group @ Botanic Garden Freiburg, University of Freiburg, D-79104 Freiburg, Germany; thomas.speck@biologie.uni-freiburg.de; 2 Cluster of Excellence *liv*MatS @ FIT—Freiburg Center for Interactive Materials and Bioinspired Technologies, University of Freiburg, D-79110 Freiburg, Germany

## 1. Introduction

During the last few decades, biomimetics has attracted increasing attention in both basic and applied research and in various fields of industry and building construction. Biomimetics has a high innovation potential and opens up possibilities for the development of innovative technical products and production chains. The specific structures and functions that the vast number of organisms have evolved in adaptation to differing environments represent the basis for all biomimetic R&D projects. Novel sophisticated methods for quantitative analyses and simulations of the form–structure–function relationship at various hierarchical levels provide intriguing insights into multiscale mechanics and other functions of biological materials and surfaces. For the first time, it is possible to transfer biological structures and thus their properties into innovative biomimetic products by means of newly developed production methods and at reasonable cost.

Animals, with their fascinating behaviour and movement processes, have long attracted interest, with plants only more recently having also been recognised as valuable concept generators for biomimetic research [1,2]. In general, from a material scientist’s point of view, plants can be regarded as fibre-reinforced materials systems defined by a number of material properties [3]. These plant materials systems consist of various components with different material properties and, thus, are not only anatomically inhomogeneous and mechanically anisotropic, but also possess a spatial and temporal heterogeneity attributable to growth and their capacity to respond or adapt to changing environmental conditions.

## 2. Broad Spectrum of Topics

The articles published in this Special Issue, “Bridging the Gap: From Biomechanics and Functional Morphology of Plants to Biomimetic Developments”, cover theoretical considerations concerning the challenges of the biomimetic approach and potential pitfalls and include the entire development chain from basic research in the field of biomechanics and functional morphology of plants to simulations and the development of physical models for a better understanding of functional principles, finally leading to biomimetic products on the laboratory scale or demonstrator level. The size scale includes all hierarchical levels from microstructure to the entire plants.

Niklas and Walker [4] detail the considerable challenges of the biomimetic approach, which uses approaches to model and abstract the behaviour or properties of living systems based on inference within structure–function relationships for the development of synthetic bioinspired structures and systems. The authors discuss potential pitfalls by comparing the ways in which engineering and biological systems are analysed operationally, address the challenges of modelling biological systems, and suggest some methods for assessing the validity of these models.

In the second contribution, Wunnenberg et al. [5] describe the strengthening structures in the petiole-lamina transition zone of peltate leaves, in which the petiole is centrally attached to the lamina. In such peltate leaves, which are found in 357 plant species, the transition from the rod-like petiole to plate-like lamina is characterised by a marked change in geometry. The authors have analysed the connection between petiole and lamina in 41 species, with a particular focus on the reinforcing fibre strands. They have discerned several design principles that can be used as models for plant-inspired lightweight supporting structures.

Masselter et al. [6] present models for 3D reticulated actuator systems inspired by the macroscopic cortical fibre networks found in some extant and fossil plants. The asymmetric deformation of these networks caused by asymmetric secondary wood growth enable the up-righting of inclined balsa and papaya stems. This functional movement principle has been transferred to elastic technical hollow tubes that are surrounded by a net-like structure. In addition, the influence of fibre angles on deformation behaviour under internal pressure is analysed and described.

Klang and Nickel [7] describe the development of biomimetic freeze-casted graded ceramics inspired by the spines of the lance sea urchin. These spines possess a plant-like hierarchical lightweight construction and represent superstructures with several gradation features including porosity, pore orientation and pore size. The spines have considerable biomimetic potential for porous ceramics with predetermined breaking points and adaptable behaviour under compression up to failure. Some of these features can be included in an abstracted way in ceramics manufactured by freeze-casting.

Poppinga et al. [8] show that a variety of complex plant movement principles can be demonstrated with comparably simple handcrafted compliant systems based on paper, wood, plastic foil and/or glue as construction materials. The handcrafted systems are self-actuated by shrinking processes triggered by the anisotropic hygroscopic properties of the wood or paper. The developed systems have a high potential for fast, precise and low-cost abstraction and transfer processes in biomimetics and for the “hands-on understanding” of plant movements in university and school courses.

Mühlich et al. [9] have analysed, through simulation and physical testing, the influence of several design criteria, such as stiffness and hinge width, in compliant folding mechanisms moved by bioinspired pneumatically actuated hinges composed of fibre-reinforced plastic. The authors have developed a workflow within a finite element model software that allows mathematical models to be inferred for the prediction of mechanical properties and of deformation behaviour as a function of the parameters used as design criteria.

Krüger et al. [10] have developed a material design space for 4D-printed bioinspired bilayer structures with hygroscopic actuation that takes into account unequal effective layer widths and deflections under self-weight. The curvature of various bilayer strips has been described using an adapted Timoshenko model, and its ability to predict curvatures has been validated in experiments. This approach has led to an analytical solution space enabling the quantification of the influence of Young’s moduli, swelling strains and densities on deflection under self-weight and curvature under hygroscopic swelling. In addition, it allows the selection of a suitable material combination in bioinspired bilayer systems with unequal layer widths.

The articles published in this Special Issue, “Bridging the Gap: From Biomechanics and Functional Morphology of Plants to Biomimetic Developments”, thus, give an up-to-date overview of current research topics in plant-inspired biomimetic research.
